# Effectiveness and ecotoxicity of zero-valent iron nanoparticles during rhizoremediation of soil contaminated with Zn, Cu, Cd and diesel

**DOI:** 10.1016/j.dib.2017.12.049

**Published:** 2018-01-03

**Authors:** Rafael G. Lacalle, María T. Gómez-Sagasti, Unai Artetxe, Carlos Garbisu, José M. Becerril

**Affiliations:** a Department of Plant Biology and Ecology, University of the Basque Country (UPV/EHU), P.O. Box 644, E-48080 Bilbao, Spain; b NEIKER, Department of Conservation of Natural Resources, c/Berreaga 1, E-48160 Derio, Spain

## Abstract

The remediation of soils simultaneously contaminated with organic and inorganic compounds is still a challenging task. The application of metallic nanoparticles, such as zero-valent iron nanoparticles (nZVI), for soil remediation is highly promising, but their effectiveness and potential ecotoxicity must be further investigated. In addition, the performance of nZVI when combined with other remediation strategies is a topic of great interest. Here, we present data on soil chemical (pseudo-total and CaCl_2_-extractable metal concentrations; petroleum hydrocarbon concentrations) and biological properties (microbial properties and phytotoxicity) after the application of nZVI to soil simultaneously contaminated with Zn, Cu, Cd and diesel, in the absence and presence of other remediation treatments such as the application of an organic amendment and the growth of *Brassica napus* plants. Soils were artificially contaminated with the abovementioned contaminants. Then, after an aging period of one month, nZVI were applied to the soil and, subsequently, *B. napus* seeds were sown. Plants were left to grow for one month. Soil samples were collected immediately after artificially contaminating the soil (T1), at sowing (T2) and at harvesting (T3). Overall, the application of nZVI had no effect on contaminant removal, nor on soil microbial parameters. In contrast, it did cause an indirect toxic effect on plant root elongation due to the interaction of nZVI with soil organic matter. These data are useful for researchers and companies interested in the effectiveness and ecotoxicity of zero-valent iron nanoparticles during the remediation of soil contaminated with metals and hydrocarbons, especially when combined with Gentle Remediation Options.

**Specifications Table**

**Table:** 

Subject area	*Environmental Sciences, Plant Sciences*
More specific subject area	*Soil ecotoxicity, nanoparticles, bioremediation*
Type of data	*Tables*
How data was acquired	*Data collected from an experiment on nZVI-assisted rhizoremediation of mixed contaminated soil*
Data format	*Analyzed*
Experimental factors	*nZVI were applied to soils with combinations of the following factors: mixed contamination (metals and diesel), organic amendment, Brassica napus plants*
Experimental features	*Analysis of pseudo-total and extractable Zn, Cu and Cd concentrations, petroleum hydrocarbon concentrations; biomass, activity and functional diversity of soil microbial communities; soil phytotoxicity*
Data source location	*Leioa, Spain (43.329456, -2.969329)*
Data accessibility	*Data are available in the article*
Related research article	*R.G. Lacalle, M.T. Gómez-Sagasti, U. Artetxe, C. Garbisu, J.M. Becerril, Brassica napus has a key role in the recovery of the health of soils contaminated with metals and diesel by rhizoremediation, Sci. Total. Environ. (2017) (In press)*

**Value of the data**

• Data show the lack of effectiveness of nZVI for the assisted rhizoremediation of soils contaminated with metals and diesel.
• Data reveal the ecotoxicity of nZVI to plants, mediated by their interaction with soil organic matter.
• Data are useful for the design of soil remediation strategies using nZVI nanoparticles.

## 1 Data

Data provided here were generated during an experiment carried out to study the effectiveness of nZVI for the remediation of mixed contaminated soils. Besides, their potential toxicity for plants and soil microbial communities was investigated. Finally, these data supplement our study on the recovery of the health of soils contaminated with metals and diesel by rhizoremediation [1].

### 1.1 Chemical parameters

Table 1 shows the characterization of the organic amendment used in this experiment. This characterization showed a high content of total organic matter, a high C/N ratio, moderate levels of some metals (Cu, Zn), and the absence of *Salmonella* spp. and *Escherichia coli*. Table 2 shows pseudo-total (A-C) and CaCl_2_-extractable (D-F) metal concentrations in soil. Pseudo-total concentrations decreased along the experiment. Zinc and Cu concentrations in amended soil were higher, compared to non-amended controls, due to the presence of these metals in the amendment itself. nZVI application had no effect on pseudo-total metal concentrations. CaCl_2_-extractable metal concentrations meant a very small fraction of pseudo-total metal concentrations, and decayed over the experimental period, especially for Zn. Similarly, the presence of Cu and Zn in the amendment itself contributed to the higher values of CaCl_2_-extractable metal concentrations observed in amended soils. CaCl_2_-extractable metal concentrations were similar in the absence *versus* presence of nZVI. Table 3 shows the concentration of total petroleum hydrocarbons-TPH (A), as well as that of the different fractions: n-alkanes (B); fatty acid methyl esters-FAME (C); alkane fraction n-C13-n-C16 (D); alkane fraction n-C17-n-C21 (D); alkane fraction n-C22-n-C30 (D), for treatments with and without nZVI. Degradation was more accentuated for the FAME fraction (90%) than for n-alkanes (60%). Degradation was faster in the amended soil. The n-C17-C21 fraction was the most abundant and most easily degraded fraction. On the other hand, n-C22-C30 was the most recalcitrant fraction. The application of nZVI had no effect on the degradation of petroleum hydrocarbons. This lack of effectivity of nZVI on metal and hydrocarbon remediation could be partially due to the application of uncoated nanoparticles.

**Table 1:** Characterization of the organic amendment used in this experiment.

**Agronomic parameters**	
Organic matter (%)	29.6
Humidity (%)	22.6
Organic C / Organic N	13.2

**Sanitary parameters**	
*Salmonella* spp.	Absent
*Escherichia coli*	Absent

**Metal concentrations**	
Cd (mg kg^-1^)	1.3
Cu (mg kg^-1^)	241
Ni (mg kg^-1^)	25.2
Pb (mg kg^-1^)	57.2
Zn (mg kg^-1^)	368
Hg (mg kg^-1^)	0.6
Cr (mg kg^-1^)	32

**Table 2:** Pseudo-total (A-C) and CaCl_2_-extractable (D-F) metal concentrations (mg kg^-1^) in soil for the different sampling times (T1-T3). Different letters indicate statistical significance (*P* < 0.05) between treatments, and numbers indicate statistical significance (*P* < 0.05) between sampling times. Asterisks refer to statistical significance (*P* < 0.05) between homologous treatments treated with and without nZVI.

	**UMN**	**UMP**	**AMN**	**AMP**	**nUMN**	**nUMP**	**nAMN**	**nAMP**
**(A) [Zn]**_**T**_								
T1	1805.4 ± 34.7 b1		2234.6 ± 88.3 a12		1805.4 ± 34.7 b1		2234.6 ± 88.3 a1	
T2	1796.2 ± 16.9 b1		2025.1 ± 22.5a1		1878.5 ± 28.9 a1		1925.2 ± 18.8 a1*	
T3	1593.8 ± 9.44 b2	1615.1 ± 3.9 b	1846.7 ± 23.4 a2	1849.0 ± 107.6 ab	1406.4 ± 23.3 c2*	1479.2 ± 28.8 c*	1893.0 ± 48.6 b1	2073.4 ± 43.0 a

**(B) [Cu]**_**T**_								
T1	757.6 ± 19.6 b1		956.1 ± 48.5 a1		757.6 ± 19.6 b1		956.1 ± 48.5 a1	
T2	690.8 ± 5.9 b1		855.5 ± 13.6 a1		771.8 ± 55.8 a1		802.6 ± 14.5 a1	
T3	666.1 ± 29.9 b1	668.6 ± 19.2 b	895.7 ± 24.8 a1	840.5 ± 46.7 a	610.6 ± 13.9 b1	605.8 ± 19.9 b	894.4 ± 23.7 a1	946.4 ± 35.5 a

**(C) [Cd]**_**T**_								
T1	62.2 ± 1.4 a1		73.6 ± 4.6 a1		62.2 ± 1.45 a1		73.6 ± 4.6 a1	
T2	65.8 ± 3.2 a1		63.7 ± 2.0 a1		67.5 ± 2.0 a1		59.0 ± 1.2 b2	
T3	62.8 ± 1.3 a1	61.0 ± 1.5 a1	65.7 ± 0.6 a1	60.4 ± 1.5 a1	61.4 ± 2.6 a1	58.1 ± 1.7 a	63.1 ± 3.1 a12	64.8 ± 1.7 a

**(D) [Zn]**_**E**_								
T1	2.0 ± 0.1 b1		4.8 ± 0.8 a1		2.0 ± 0.1 b1		4.8 ± 0.8 a1	
T2	0.5 ± <0.1 ab2		0.7 ± <0.1 a2		0.4 ± <0.1 b2		0.5 ± <0.1 ab2	
T3	0.3 ± <0.1 b3	0.3 ± <0.1 b	0.4 ± 0.1 a2	0.4 ± <0.1 a	0.3 ± <0.1 b3	0.3 ± <0.1 a*	0.3 ± <0.1 a2	0.3 ± <0.1 a*

**(E) [Cu]**_**E**_								
T1	0.08 ± <0.1 b1		0.3 ± <0.1 a1		0.1 ± <0.1 b1		0.3 ± <0.1 a1	
T2	0.1 ± <0.1 b1		0.2 ± <0.1 a2		<0.1 ± <0.1 b1		0.3 ± <0.1 a1	
T3	0.1 ± <0.1 b2	0.1 ± <0.1 b	0.1 ± <0.1 a3	0.2 ± <0.1 a	0.1 ± <0.1 c1*	0.1 ± <0.1 c*	0.2 ± <0.1 b2*	0.2 ± <0.1 a*

**(F) [Cd]**_**E**_								
T1	0.2 ± <0.1 a1		0.15 ± <0.1 a		0.2 ± <0.1 a1		0.2 ± <0.1 a1	
T2	0.1 ± <0.1 a2		<0.1 ± <0.1 b2		0.1 ± <0.1 a2		<0.1 ± <0.1 a2	
T3	<0.1 ± <0.1 a3	<0.1 ± <0.1 a	<0.1 ± <0.1 b12	<0.1 ± <0.1 b	<0.1 ± <0.1 a2	<0.1 ± <0.1 a	<0.1 ± <0.1 b2	<0.1 ± <0.1 b

**Table 3:** Concentrations (mg kg^-1^) of TPH (Total Petroleum Hydrocarbons, A); n-Alk (n-alkanes, B); FAMEs (Fatty Acid Methyl Esters, C); and n-alkane fractions (D-F) in soil for the different sampling times (T1-T3). Different letters indicate statistical significance (*P* < 0.05) between treatments, and numbers indicate statistical significance (*P* < 0.05) between sampling times. Asterisks refer to statistical significance (*P* < 0.05) between homologous treatments treated with and without nZVI.

	**UMN**	**UMP**	**AMN**	**AMP**	**nUMN**	**nUMP**	**nAMN**	**nAMP**
**(A) TPH**								
T1	2860.5 ± 117.7 a1		2417.3 ± 175.6 a1		2860.5 ± 117.7 a1		2417.3 ± 175.6 a1	
T2	1631.5 ± 46.1 a2		997.4 ± 25.6 b2		1467.5 ± 131.8 a2		793.5 ± 32.4 b2*	
T3	888.6 ± 15.1 a3	933.8 ± 23.3 a	905.5 ± 21.2 a2	816.7 ± 59.5 a	1089.8 ± 49.7 a3*	883.9 ± 189.4 a	831.9 ± 81.3 a2	883.9 ± 113.1 a

**(B) n-Alk**								
T1	2648.2 ± 109.3 a1		2231.0 ± 163.2 a1		2648.2 ± 109.3 a1		2230.9 ± 163.2 a1	
T2	1608.4 ± 47.3 a 2		975.0 ± 33.4 b2		1440.9 ± 126.0 a2		766.8 ± 29.3 b2*	
T3	874.9 ± 15.8 a3	924.6 ± 23.0 a	891.0 ± 20.2 a2	804.4 ± 60.6 a	1082.9 ± 49.3 a3*	858.9 ± 185.5 a	815.3 ± 81.2 a2	867.0 ± 111.6 a

**(C) FAME**								
T1	209.5 ± 8.6 a1		176.5 ± 12.9 a1		209.5 ± 8.6 a1		176.5 ± 12.9 a1	
T2	13.5 ± 0.2 a2		3.3 ± 0.4 b2		9.7 ± 1.2 a2*		2.7 ± 0.2 b2	
T3	3.6 ± 0.4 a3	4.5 ± 0.2 a	3.9 ± 0.6 a2	5.2 ± 1.6 a	5.5 ± 0.4 a2	3.6 ± 1.3 a	3.3 ± 0.1 a2	3.7 ± 0.4 a

**(D) n-C13**–**n-C16**								
T1	662.8 ± 37.3 a1		510.4 ± 44.7 a1		662.8 ± 37.3 a1		510.4 ± 44.7 a1	
T2	288.0 ± 4.1 a2		196.1 ± 6.2 b2		280.0 ± 26.9 a2		164.5 ± 13.1 b2	
T3	194.0 ± 21.3 a3	227.6 ± 20.3 a	200.1 ± 9.8 a2	184.5 ± 17.6 a	240.9 ± 9.0 a2	184.7 ± 48.3 a	175.1 ± 23.7 a2	190.5 ± 37.5 a

**(E) n-C17**–**n-C21**								
T1	1190.2 ± 26.2 a1		1046.2 ± 70.0 a1		1190.2 ± 26.2 a1		1046.2 ± 70.0 a1	
T2	816.6 ± 40.2 a2		386.7 ± 30.7 b2		677.7 ± 73.5 a2		266.9 ± 7.0 b2	
T3	314.2 ± 33.0 a3	312.7 ± 9.0 a	275.7 ± 6.9 a2	278.8 ± 30.3 a	431.6 ± 18.7 a3*	319.5 ± 87.4 a	262.6 ± 17.2 a2	295.1 ± 35.7 a

**(F) n-C22**–**n-C30**								
T1	783.3 ± 44.8 a1		657.2 ± 58.9 a1		783.3 ± 44.8 a1		657.2 ± 58.9 a1	
T2	488.1 ± 14.5 a2		367.6 ± 7.7 b2		460.1 ± 29.5 a2		316.3 ± 15.0 b2*	
T3	351.2 ± 36.3 a3	374.1 ± 5.9 a	398.3 ± 23.6 a2	321.9 ± 16.1 a	399.7 ± 22.9 a2	340.0 ± 50.0 a	353.4 ± 43.7 a2	355.1 ± 34.9 a

### 1.2 Biological parameters

Values of soil microbial properties (BS-basal respiration, SIR-substrate induced respiration, AWCD-average well color development, NUS-number of used substrates) in the absence and presence of nZVI are shown in Table 4 and Table 5, respectively. Overall, values of BR and SIR were higher in the presence of the amendment. Basal respiration increased in soils exposed to the mixed contamination, while SIR values were similar to those observed in control soil. nZVI application did not have any significant effect on these respiratory parameters. Regarding soil microbial functional diversity, AWCD and NUS values were not affected by the application of the amendment nor by the presence of the contaminants, but they were highly stimulated by the presence of *B. napus* plants. The application of nZVI did not affect the soil microbial functional diversity. Table 6 shows data from the root elongation bioassay performed with *Cucumis sativus* in soils treated with (A) and without (B) nZVI. A general trend towards decreased root elongation values in the presence of contaminants and increased values in the presence of the amendment was identified. The application of nZVI caused an indirect toxic effect on plant root elongation due to the interaction of nZVI with soil organic matter. This interaction of nZVI and soil organic matter needs further investigation.

**Table 4.** Soil microbial properties in the absence of nZVI. BR: Basal Respiration (µg CO_2_ g^-1^ DW soil h^-1^); SIR: Substrate Induced Respiration (µg CO_2_ g^-1^ DW soil h^-1^); AWCD: Average Well Color Development; NUS: Number of Used Substrates. Sampling times = T0-T3. Different letters indicate statistical significance (*P* < 0.05) between treatments, and numbers indicate statistical significance (*P* < 0.05) between sampling times.

	**UCN**	**UCP**	**UMN**	**UMP**	**ACN**	**ACP**		**AMN**	**AMP**
**BR**									
T0	0.9 ± 0.1 b1				1.9 ± 0.3 a1				
T1	1.6 ± 0.2 b1				2.8 ± 0.4 a2				
T2	1.3 ± 0.1 d1		2.7 ± 0.1 c1		3.5 ± <0.1 b2			4.7 ± 0.2 a1	
T3	1.3 ± 0.2 e1	1.6 ± 0.1 e	2.7 ± 0.2 d1	2.8 ± 0.1 cd	3.5 ± 0.1 bc2	4.1 ± 0.1 b		4.4 ± 0.1 b1	5.7 ± 0.3 a

**SIR**									
T0	5.7 ± 0.2 a1				6.8 ± 0.5 a1				
T1	8 ± 0 a2				8.1 ± 0.1 a12				
T2	5.2 ± <0.1 b1		6.7 ± 1.1 b1		11.3 ± 0.4 a3			11.2 ± 1.1 a1	
T3	8.6 ± 0.7 b2	8.4 ± 0.7 b	7.8 ± 1.1 b1	8.3 ± 1.1 b	15.3 ± 1.4 a23		13.2 ± 1.8 ab	10.0 ± 0.7 ab1	11.5 ± 1.8 ab

**AWCD**									
T0	1.0 ± 0.2 a1				0.8 ± 0.1 a1				
T1	0.8 ± 0.1 a2				0.9 ± 0.2 a1				
T2	0.2 ± <0.1 a1		0.2 ± 0.1 a1		0.3 ± 0.1 a2			0.2 ± <0.1 a1	
T3	0.3 ± <0.1 bcd2	0.4 ± <0.1 b	0.2 ± <0.1 cd1	0.2 ± <0.1 d	0.3 ± 0.1 bc2		0.6 ± <0.1 a	0.2 ± <0.1 cd1	0.7 ± <0.1 a

**NUS**									
T0	25.8 ± 2.3 a1				21.1 ± 1.2 a1				
T1	19.0 ± 1.2 a2				20.3 ± 1.3 a1				
T2	6.4 ± 0.3 a3		3.6 ± 0.4 b1		5.8 ± 0.4 a2			6.3 ± 0.2 a1	
T3	8.4 ± 1.3 bc3	10.5 ± 1.2 b	7.7 ± 0.8 bc2	7.0 ± 0.7 bc	8.5 ± 1.0 bc2		16.5 ± 0.4 a	5.6 ± 0.9 c1	17.5 ± 0.7 a

**Table 5:** Soil microbial properties in the presence of nZVI. BR: Basal Respiration (µg CO_2_ g^-1^ DW soil h^-1^); SIR: Substrate Induced Respiration (µg CO_2_ g^-1^ DW soil h^-1^); AWCD: Average Well Color Development; NUS: Number of Used Substrates. Sampling times = T0–T3. Different letters indicate statistical significance (*P* < 0.05) between treatments, and numbers indicate statistical significance (*P* < 0.05) between sampling times (T0-T3). Asterisks refer to statistical significance (*P* < 0.05) between homologous treatments treated with and without nZVI.

	**nUCN**	**nUCP**	**nUMN**	**nUMP**	**nACN**			**nACP**	**nAMN**	**nAMP**
**BR**										
T0	0.9 ± <0.1 b1				1.9 ± 0.3 a1					
T1	1.6 ± 0.2 b2				2.8 ± 0.4 a12					
T2	1.4 ± 0.1 c2		2.4 ± 0.1 b1		2.9 ± 0.1 b2*				3.9 ± 0.3 a1	
T3	1.5 ± 0.2 e2	1.8 ± 0.2 e	3.0 ± 0.1 d2	2.7 ± <0.1 d	3.9 ± 0.3 c3	4.2 ± 0.3 bc			4.7 ± 0.2 b1	5.9 ± 0.2 a

**SIR**										
T0	5.7 ± 0.2 a1				6.8 ± 0.5 a1					
T1	8.0 ± <0.1 a2				8.1 ± 0.1 a1					
T2	10.0 ± 0.7 a12*		10.8 ± 0.4 a1*		12.3 ± 1.7 a12				14.12 ± 0.4 a1	
T3	9.4 ± 1.0 b12	9.0 ± 1.3 b	7.4 ± 0.7 b2	9.6 ± 1.0 b	15.1 ± 0.9 a2	18.2 ± 2.5 a			14.4 ± 1.4 a1*	14.6 ± 0.7 a

**AWCD**										
T0	1.0 ± 0.2 a1				0.8 ± 0.1 a1					
T1	0.8± 0.1 a1				1.0 ± 0.2 a1					
T2	0.2 ± 0.1 a2		0.2 ± <0.1 a1		0.3 ± <0.1 a2				0.2 ± 0.1 a1	
T3	0.2 ± <0.1 c2	0.3 ± <0.1 b	0.1 ± <0.1 c1	0.1 ± <0.1 c	0.3 ± <0.1 b2		0.7 ± <0.1 a		0.1 ± <0.1 c1	0.8 ± <0.1 a

**NUS**										
T0	25.8 ± 2.3 a1				21.1 ± 1.2 a1					
T1	19.0 ± 1.2 a2				20.3 ± 1.3 a1					
T2	8.6 ± 1.0 a3		4.6 ± 0.6 b1		8.6 ± 1.0 a2*				6.3 ± 1.7 ab1	
T3	6.2 ± 1.0 b3	9.1 ± 1.7 b	6.3 ± 1.3 b1	6.0 ± 1.9 b	8.8 ± 1.5 b2	16.6 ± 0.8 a			6.7 ± 1.1 b1	19.1 ± 0.6 a

**Table 6:** Root Elongation (RE, mm) of *Cucumis sativus* seedlings exposed to treatments without nZVI (A) and with nZVI (B) at T2 and T3 sampling times. Different letters indicate statistical significance (*P* < 0.05) between treatments, and numbers indicate statistical significance (*P* < 0.05) between sampling times. Asterisks refer to statistical significance (*P* < 0.05) between homologous treatments treated with and without nZVI.

	**UCN**	**UCP**	**UMN**		**UMP**	**ACN**	**ACP**	**AMN**	**AMP**
**(A) RE without nZVI**									
T2	20.1 ± 0.9 a1			8.2 ± 0.6 b1		24.8 ± 1.1 b1		34.7 ± 2.6 a1	
T3	41.1 ± 0.6 a2	40.4 ± 0.8 a		20.6 ± 2.9 c2	16.8 ± 1.3 c	51.1 ± 1.6 bc2	49.5 ± 2.3 c	55.1 ± 0.5 ab2	56.7 ± 1.6 a
								
	**nUCN**	**nUCP**		**nUMN**	**nUMP**	**nACN**	**nACP**	**nAMN**	**nAMP**

**(B) RE with nZVI**									
T2	19.0 ± 3.2 a1			8.2 ± 0.3 b1		15.2 ± 2.1 c1*		27.5 ± 1.8 b1*	
T3	46.8 ± 0.5 a2	46.0 ± 1.9 a2		21.5 ± 2.1 c2	28.8 ± 4.5 b2	46.7 ± 1.4 cd2	42.9 ± 3.0 d*	49.2 ± 0.7 c2*	48.8 ± 1.0 c

## 2 Experimental design, materials and methods

Two topsoils were collected (Time = T0) from a peri-urban area: one amended with 100 t ha^-1^ of an organic material produced from the recycling of urban organic wastes, and the other without such amendment. The organic amendment was obtained from the “BIOCOMPOST DE ALAVA” company, an urban waste treatment plant. After selective separation and sieving, organic matter from domestic waste of the city of Vitoria-Gasteiz (Spain) was stored for 6 months before use. Soil was sieved to <6 mm, air-dried, and half of each soil was artificially contaminated with a mixture of metals and commercial diesel fuel purchased from a petrol station (T1). Experimental metal concentrations were (in mg kg^-1^ DW soil): Zn (1500), Cu (500) and Cd (50). Immediately after, diesel (6000 mg kg^-1^ DW soil) was added to already metal contaminated soils, following ISO 15952 [2]. Then, 700 g DW of contaminated or non-contaminated soil were placed in 1 L pots. In order to allow contaminant stabilization, pots were kept for one month in a phytotron under the following controlled conditions: photoperiod 14/10 h day/night, temperature 25/18 ^o^C day/night, relative humidity 60/80% day/night, and a photosynthetic photon flux density of 200 µmol photon m^-2^ s^-1^. After the 1-month stabilization period (T2), nZVI (NanoFer Star, Nanoiron s.r.o) were activated following manufacturer's instructions with Milli-Q water for 24 h and then applied in aqueous solution to half of the pots (contaminated and non-contaminated) at a concentration of 1 g nZVI kg^-1^ DW soil. Three days later, *Brassica napus* seeds were sown on half of the pots, and plants were harvested a month later (T3). Soil samples were collected at spiking (T1), sowing (T2) and harvesting time (T3).

Contaminant concentrations were measured in the collected soil samples. In order to measure pseudo-total Zn, Cu and Cd concentrations, samples were digested according to US-EPA Method 3051A [3]. For extractable metal concentrations, an extraction was performed following Houba et al. [4]. Metal concentrations were quantified by Inductively-Coupled Plasma Mass Spectrometry (Agilent 7700). Total petroleum hydrocarbon and fatty acid methyl ester concentrations in soil were measured by Gas Chromatography–Mass Spectrometry (GC–MS), as described in Bartolomé et al. [5]. Soil samples were used to determine the following microbial properties, as detailed in Galende et al. [6]: (i) microbial activity was determined by basal respiration (BR) following ISO 16072 [7]; (ii) potentially active microbial biomass was determined by substrate-induced respiration (SIR) following ISO 17155 [8]; (iii) average well color development (AWCD) and (iv) number of metabolized substrates (NUS) were determined from Biolog EcoPlates^TM^ following Epelde et al. [9]. A root elongation bioassay with *Cucumis sativus* was performed to determine soil phytotoxicity. Seeds of *C. sativus* (c.v. Marketmore) were pre-germinated on Petri dishes, containing wet filter paper, for 3 days under controlled conditions (14/10 h day/night; 25/18 ^o^C day/night; and full darkness). Concurrently, 10 g of dried soil were placed on Petri dishes, hydrated with deionized water, mixed vigorously, and covered with filter paper. After pre-germination, seven seeds of *C. sativus* showing a radicle length of 5–10 mm were placed over the filter paper of soil-containing Petri dishes. Afterwards, dishes were placed for 72 h under the following conditions: photoperiod 14/10 h day/night, temperature 25/18 ^o^C day/night, relative humidity 60/80% day/night, and photosynthetic photon flux density of 100 µmol photon m^-2^ s^-1^. Images of the seedlings were taken at the beginning and after 72 h of incubation with the soil. Images were processed by ImageJ Software. Root Elongation (RE) (RE = RE_T2_ – RE_T1_) was calculated for each seedling. Data were statistically analyzed using ANOVA-test when data were normally distributed and Kruskal-Wallis test when they were not. Kolmogorov-Smirnov was used as normality test.

Samples were identified according to the following codes:

**Table:** 

	Unamended (U)				Amended (A)			
	Control (C)		Mixed contamination (M)		Control (C)		Mixed contamination (M)	
	Without nZVI	With nZVI (n)	Without nZVI	With nZVI (n)	Without nZVI	With nZVI (n)	Without nZVI	With nZVI (n)
Not planted	UCN	nUCN	UMN	nUMN	ACN	nACN	AMN	nAMN
Planted	UCP	nUCP	UMP	nUMP	ACP	nACP	AMP	nAMP
